# Supplementary material for: 0.1% RGN-259 (Thymosin ß4) Ophthalmic Solution Promotes Healing and Improves Comfort in Neurotrophic Keratopathy Patients in a Randomized, Placebo-Controlled, Double-Masked Phase III Clinical Trial
Source: Int J Mol Sci. 2022 Dec 29;24(1):554. doi: 10.3390/ijms24010554 (PMC9820614; doi:10.3390/ijms24010554)
Supplement: Supplementary file 1 [file ijms-24-00554-s001.zip › ijms-2080588-supplementary.pdf]

Table S1 Shift from Baseline for Epithelial Defect Mackie Classification at Visits 2, 3, 4, 5, 6, and 7

Shift from Baseline (Improvement or No Change)	0.1% RGN-259 (N=10)	Placebo (N=8)
**Visit 2 (Day 8 ± 2)**		
Baseline Stage 2		
No Change or Worsening	8 (80.0%)	5 (62.5%)
Shift to Stage 1	1 (10.0%)	2 (25.0%)
Shift to Stage N/A	0	0
Baseline Stage 3		
No Change or Worsening	1 (10.0%)	0
Shift to Stage 2	0	1 (12.5%)
Shift to Stage 1	0	0
Shift to Stage N/A	0	0
**Visit 3 (Day 15 ± 2)**		
Baseline Stage 2		
No Change or Worsening	4 (40.0%)	5 (62.5%)
Shift to Stage 1	4 (40.0%)	2 (25.0%)
Shift to Stage N/A	1 (10.0%)	0
Baseline Stage 3		
No Change or Worsening	0	0
Shift to Stage 2	1 (10.0%)	0
Shift to Stage 1	0	1 (12.5%)
Shift to Stage N/A	0	0
**Visit 4 (Day 22 ± 2)**		
Baseline Stage 2		
No Change or Worsening	3 (30.0%)	5 (62.5%)
Shift to Stage 1	5 (50.0%)	2 (25.0%)
Shift to Stage N/A	1 (10.0%)	0
Baseline Stage 3		
No Change or Worsening	0	0
Shift to Stage 2	0	0
Shift to Stage 1	1 (10.0%)	1 (12.5%)0
Shift to Stage N/A	0	0
**Visit 5 (Day 29 ± 2)**		
Baseline Stage 2		
No Change or Worsening	2 (20.0%)	6 (75.0%)
Shift to Stage 1	5 (50.0%)	1 (12.5%)
Shift to Stage N/A	2 (20.0%)	0
Baseline Stage 3		
No Change or Worsening	0	0
Shift to Stage 2	0	0

**Shift from Baseline (Improvement or No Change)**	**0.1% RGN-259 (N=10)**	**Placebo (N=8)**
Shift to Stage 1	1 (10.0%)	1 (12.5%)
Shift to Stage N/A	0	0
**Visit 6 (Day 36 ± 3)**		
Baseline Stage 2		
No Change or Worsening	3 (30.0%)	6 (75.0%)
Shift to Stage 1	4 (40.0%)	1 (12.5%)
Shift to Stage N/A	2 (20.0%)	0
Baseline Stage 3		
No Change or Worsening	0	1 (12.5%)
Shift to Stage 2	0	0
Shift to Stage 1	1 (10.0%)	0
Shift to Stage N/A	0	0
**Visit 7 (Day 43 ± 3)**		
Baseline Stage 2		
No Change or Worsening	3 (30.0%)	6 (75.0%)
Shift to Stage 1	4 (40.0%)	1 (12.5%)
Shift to Stage N/A	2 (20.0%)	0
Baseline Stage 3		
No Change or Worsening	0	0
Shift to Stage 2	0	1 (12.5%)
Shift to Stage 1	0	0
Shift to Stage N/A	1 (10.0%)	0
